# Prognostic Nutritional Index and Neutrophil/Lymphocyte Ratio Can Serve as Independent Predictors of the Prognosis of Hepatocellular Carcinoma Patients Receiving Targeted Therapy

**DOI:** 10.1155/2022/1389049

**Published:** 2022-08-11

**Authors:** Wei Chen, Mingjun Zhang, Chanjuan Chen, Xiaonan Pang

**Affiliations:** Department of Oncology, Second Affiliated Hospital of Anhui Medical University, Hefei 230601, Anhui, China

## Abstract

**Objective:**

The prognostic nutritional index (PNI) is an immunonutritional indicator, and the neutrophil/lymphocyte ratio (NLR) reflects the inflammatory status. This research intends to determine the implications of NLR and PNI in evaluating the outcome of hepatocellular carcinoma (HCC) patients undergoing targeted therapy (TT).

**Methods:**

We retrospectively analyzed 83 patients' records with sorafenib treatment for advanced HCC in the Second Affiliated Hospital of Anhui Medical University. Patient records comprised general data and blood routines. The PNI and NLR values were calculated using the serum albumin levels (ALB), neutrophil (NEU) count, and lymphocyte (LY) count. The optimal thresholds of the PNI and NLR for predicting HCC patients' outcomes were calculated by X-tile. Patients were further assigned to low- and high-groups of PNI and NLR according to their thresholds. By using the Cox proportional hazards regression models, univariate and multivariate analyses were conducted to identify risk factors influencing the patient's prognosis.

**Results:**

The participants were assigned to the corresponding low-PNI (≤42.9; *n* = 10) and high-PNI (>42.9; *n* = 73) groups, as well as low-NLR (≤2.4; *n* = 64) and high-NLR (>2.4; *n* = 19) groups based on the critical values of PNI (42.9) and NLR (2.4) obtained through the X-tile calculation. A higher overall survival (OS) rate was observed in the high-PNI group and low-NLR group, than in the low-PNI group and high-NLR group, respectively. The disease control rate showed no evident difference between the groups. The PNI and NLR were of high reliability in predicting the OS of patients. Cox multivariate analysis identified the independence of the PNI and NLR as prognostic factors for patients receiving TT for advanced HCC.

**Conclusions:**

The pretreatment PNI and NLR levels have great prognostic implications for advanced HCC patients receiving TT. A higher PNI and a lower NLR suggest a higher postoperative survival rate.

## 1. Introduction

Primary liver cancer (LC) ranks 6th in incidence among the neoplastic diseases and 3rd in cancer mortality [1], inflicting nearly 906,000 new cases and causing 830,000 deaths in the year 2020 [2]. The main risk factors of LC vary from region to region, with aflatoxin exposure, chronic hepatitis B virus (HBV) infection, or both being the key decision factors in most at-risk districts (China, South Korea, and sub-Saharan Africa) [2]. In addition, excessive drinking is also one of the risk factors [3]. The primary LC has several subtypes, among which HCC (HCC) is the most prevalent, accounting for 78% of all LC cases [1]. Currently, surgery is still recognized as the most effective means to treat early and middle-stage HCC. However, due to the characteristics of insidious onset, rapid development, and high degree of malignancy, more than 70% of the cases are diagnosed at advanced stages when surgery is not applicable, with a postoperative recurrence rate as high as 70% [4].

Transcatheter arterial chemoembolization (TACE) based interventional therapy [5] is mainly used for advanced HCC patients, but it is not so effective in improving patients' survival and life quality [6]. As clinical research advances, molecularly targeted agents such as sorafenib, lenvatinib, stivarga, bevacizumab, and ramucirumab have opened a new chapter in the systemic treatment of advanced HCC, which can prolong patient survival to a certain extent [7–11]. However, regarding the prediction of HCC patients' outcomes, the conventional prognostic indices such as tumour size, blood vessel invasion, and other surgical resection-related factors [12] are less convenient in clinical testing, with limited ability to predict patient outcomes after the targeted therapy (TT). Therefore, it is necessary to find novel indices with the prognostic utility to assist in predicting the clinical outcomes of HCC patients after TT. Existing research indicates that systemic inflammatory response and systemic nutritional status indicators are critical in the prognostic risk assessment of malignant tumors [13]. Besides, related studies have shown that inflammation directly affects tumor cell growth. Inflammation benefits tumour formation, progression, and metastasis, and chronic inflammation has an association with an elevated cancer risk [14, 15]. Immune status, including the nutritional status and inflammation state, is important for the survival of patients with cancer of various types, including HCC. With the deepening of people's understanding of tumor-related inflammation, biomarkers of systemic inflammation like C-reactive protein (CRP) level, as well as the ratio of neutrophil/lymphocyte (NLR), lymphocyte-to-monocyte (LMR), and platelet-to-lymphocyte (PLR), have been well-documented to be able to predict the prognosis of neoplastic diseases, including lung carcinoma [16], gastric carcinoma [17], and liver carcinoma [18]. But the prognosis of tumor patients depends not only on tumor pathology and immune factors but also on the host state, such as the patient's nutritional status [19]. Evidence has shown that the incidence of malnutrition in malignant tumor patients is as high as 40%–80%, with approximately 30% of cancer deaths resulting from malnutrition and its resulting complications, rather than the tumor itself [20]. The prognostic nutritional index (PNI) was developed by Smale et al. [21] and was initially used for preoperative nutritional status and postoperative complication assessment and is calculated from the serum albumin (ALB) level and the peripheral blood (PB) and lymphocyte (LY) count. As a nutritional-immune index, it has been confirmed to be a prognostic index for various malignancies, such as gastric, pancreatic, and esophageal carcinomas [22–24]. A meta-analysis examining the correlation of PNI with tumor survival from 14 papers found that low-PNI levels were linked to adverse overall survival (OS) in terms of tumor type, operation conditions, threshold, as well as sample size and area [25]. Another research compared the PNI before and after the operation and confirmed the effectiveness of preoperative PNI in the prognosis prediction of patients after anticancer drug therapy [26]. It can also be used as a biomarker to predict therapeutic efficacy, even in immune checkpoint inhibitor (ICI) monotherapy [27].

In general, the PNI is an immunonutritional indicator, and the neutrophil/lymphocyte ratio (NLR) reflects the inflammatory status, both of which are of high accuracy, low cost, and high reproducibility with wide application in blood surveys. Therefore, the innovation of this research is to explore their implications in the prognosis evaluation of patients who received TT for advanced HCC.

## 2. Materials and Methods

### 2.1. Research Participants

A retrospective analysis of 83 advanced HCC patients' records treated between January 2018 and June 2020 in the Second Affiliated Hospital of Anhui Medical University was conducted. The male-to-female ratio, age (years old) range, as well as the mean age of patients were 71 : 12, 27–82, and 57.31 ± 11.85, respectively. Of them, 40 cases were pathologically confirmed and recurred after surgical treatment and relapsed again after TACE, and 43 cases of primary HCC were diagnosed by biopsy. Inclusion criteria: (1) diagnosis of HCC by cytology; (2) Barcelona clinic Liver Cancer (BCLC) stage [28]: B/C; (3) those unsuitable for conventional therapy or relapsed after conventional treatment and were therefore given molecular targeted drug therapy; (4) available blood routine and biochemical examination data within one week before TT; and (5) complete clinical data and follow-up. Exclusion criteria: (1) blood system or immune system diseases; (2) presence of a secondary tumor; (3) recent blood transfusions; (4) serious cardiac, pulmonary, hepatic, or renal dysfunction; and (5) Child–Pugh class C [29]. This study has obtained approval from the hospital's Ethical Committee.

### 2.2. Data Collection and Treatment Methods

Clinicopathological data of patients were collected, including sex, age, alcoholism history, BCLC stage, Eastern Cooperative Oncology Group (ECOG) score, Child–Pugh classification, as well as blood routines and biochemical and tumor marker indexes one week before treatment. Preoperative PNI = serum ALB (g/L) + 5 × PB LY count (10^9^/L) [30]. NLR = neutrophil (NEU) count (×10^9^/L)/LY count (×10^9^/L).

Treatment: all patients received oral sorafenib (400 mg/time, twice a day) [31], with every 28 days as a cycle, which was withdrawn when tumor progression or intolerable adverse reactions were observed. Other antitumor treatments were discontinued during the medication.

### 2.3. Efficacy Evaluation and Follow-Up

Efficacy evaluation [32] (complete response, CR; partial response, PR; stable disease, SD; and progressive disease, PD) was performed after ≥2 cycles of treatment by referring to the Response Evaluation Criteria In Solid Tumors (RECIST) 1.1. Disease control rate (DCR) = (CR + PR + SD) cases/total number of cases × 100%.

The follow-up was as of December 2021. The follow-up data were obtained through telephone or last hospitalization records and outpatient review results, and the OS (time from initiation of TT to the last follow-up or patient death derived from any cause) was calculated.

### 2.4. Statistical Processing

The software used for data statistical analysis was the SPSS 23.0 (IBM, New York, NY, USA). The X-tile (version 3.6.1) was used for the diagnostic thresholds of the NLR and PNI [33]. The intergroup comparison of the quantitative data (denoted as Mean ± SD) employed the *t*-test. Enumeration data was described as *n* (%), and the difference between the groups was tested by the *χ*^2^ or Fisher exact probability test. For the patient survival, the visualization, comparison as well as univariate and multivariate analyses were carried out by the Kaplan–Meier curves, the log-rank test, and the Cox proportional hazard regression models, respectively. Sensitivity, specificity, and area under the curve (AUC) were calculated by the receiver operator characteristic (ROC) curves. *P* < 0.05 was the significance level (*α* = 0.05).

## 3. Results

### 3.1. Determination of Optimal Critical Values of the PNI and NLR

**Figure 1:**
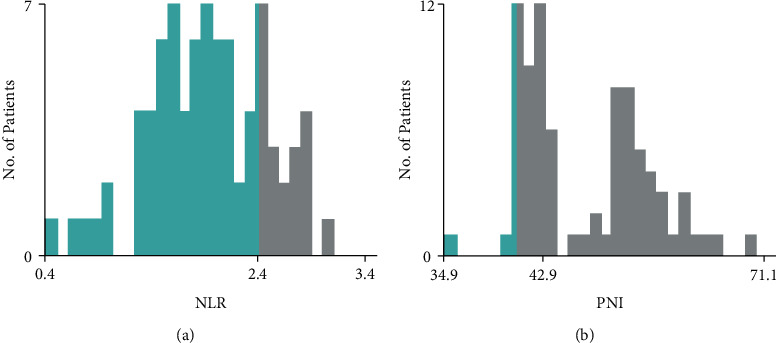
Determination of optimal critical values of the prognostic nutritional index (PNI) and neutrophil/lymphocyte ratio (NLR) by the X-tile. (a) The optimal threshold for the NLR and (b) the optimal threshold for the PNI.

The optimal cutoffs of the NLR and PLR were defined as those with the minimum *P* value of the log-rank test in the X-tile (Figure 1). Therefore, 42.9 and 2.4 were used as the optimal thresholds for the PNI and NLR, respectively, based on which, the participants were assigned to the low-PNI (≤42.9) and high-PNI (>42.9) as well as the low-NLR (≤2.4) and high-NLR (>2.4) groups.

### 3.2. Association of the Different PNI and NLR Levels with Patients' Clinicopathological Features

According to the optimal thresholds of the PNI and NLR, cases were assigned to low-PNI (*n* = 10) and high-PNI (*n* = 73) as well as low-NLR (*n* = 64) and high-NLR (*n* = 19) groups. The relationship between the PNI, NLR, and related clinicopathological features was further analyzed. As indicated in Table 1, the pretreatment level of the PNI was significantly correlated with patients' alcoholism history (*P* < 0.05) but had no obvious correlation with patients' other clinicopathological features (*P* > 0.05). While no notable association was present between the pretreatment NLR level with all clinicopathological features (*P* > 0.05; Table 2).

### 3.3. Correlation of the PNI and NLR with Short-Term Efficacy

**Figure 2:**
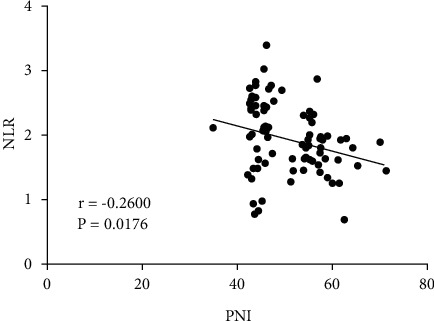
Correlation between the prognostic nutritional index (PNI) and neutrophil/lymphocyte ratio (NLR).

A significant inverse relationship between the PNI and NLR was identified by the Spearman correlation analysis (Figure 2). Short-term efficacy evaluation was completed in all patients, and we determined 24 cases of CR, 27 of PR, 9 of SD, and 23 of PD, with a DCR of 72.3%. No statistical difference was determined in the DCR between the high-PNI group and the low-PNI group (72.6% *vs* 70.0%; *χ*^2^ = 0.0122, *P*=0.9121), nor was there any statistical difference in the DCR between the high-NLR group and the low-NLR group (57.9% *vs* 76.6%; *χ*^2^ = 3.2131, *P*=0.0731), as indicated by Table 3.

### 3.4. Survival of the Different PNI and NLR Groups

**Figure 3:**
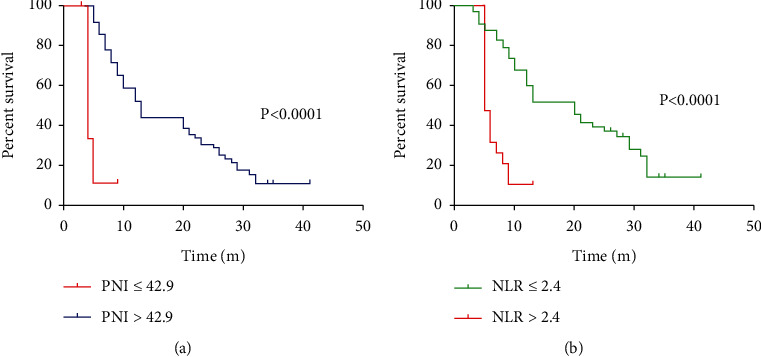
Survival analysis of the different prognostic nutritional index (PNI) and neutrophil/lymphocyte ratio (NLR) groups. (a) The Kaplan–Meier curve of the patients' overall survival rate in high- and low-PNI groups. (b) The Kaplan–Meier curve of the patients' overall survival rate in high- and low-NLR groups.

The survival status in different subgroups was analyzed using the Kaplan–Meier method, so as to determine the predictive capacity of the two for patients' OS after TT. The results showed a worse postoperative survival rate in the PNI ≤42.9 group compared with the PNI >42.9 group (*P* < 0.05; Figure 3(a)) and a better postoperative survival rate of patients in the NLR ≤2.4 group versus the NLR >2.4 group (*P* < 0.05; Figure 3(b)).

### 3.5. Predictive Utility of the PNI and NLR for Patient Prognosis

**Figure 4:**
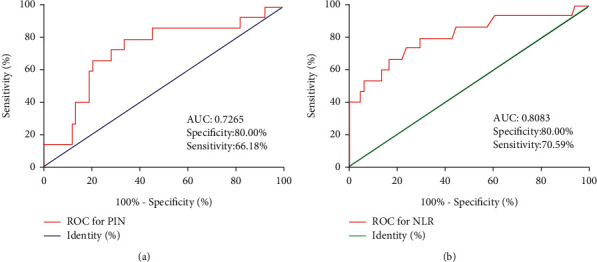
The receiver operating characteristic (ROC) analysis of sensitivity and specificity. (a) The ROC curves for the prognostic nutritional index PNI, and (b) ROC curves for the neutrophil/lymphocyte ratio NLR. *Note.* AUC: area under the curve.

The ROC analysis yielded an AUC of 0.7265 and 0.8083 based on the PNI and NLR, respectively (Figure 4), suggesting their reliability in predicting patients' OS.

### 3.6. Analysis of Related Factors Affecting Patients' OS

In the univariate regression analysis, the factors that had an obvious correlation with OS were determined to be PNI (*P* < 0.05, HR = 0.209, 95% CI: 0.114–0.382) and NLR (*P* < 0.05, HR = 2.566, 95% CI: 1.498–4.397), as shown in Table 4. Then, the two variables were included in the multivariate regression analysis, and no collinearity between PNI and NLR was confirmed by linear regression analysis. The results of multivariate regression analysis revealed the role of both PNI and NLR as independent influencing factors for patients' OS (Table 5).

## 4. Discussion

In this study, the PNI and NLR of 83 patients receiving TT for advanced HCC were analyzed. The cut-off point was determined by X-tile, based on which the participants were assigned to high or low PNI/NLR groups. The results identified an obvious association between PNI and alcoholism history of advanced HCC patients. The PNI, calculated from the serum ALB content and the total count of PB LYs, was initially developed for the assessment of perioperative nutritional status and surgical risk of patients with gastrointestinal surgery. The PNI combines two indexes, namely, the serum concentration of ALB and the total count of PB LYs. As a vital metabolic organ, the liver is responsible for the synthesis of ALB and the long-term insufficient protein intake can cause a decrease in the ALB. Hence, ALB, an indicator of chronic protein malnutrition, can be used to assist in assessing the general nutritional condition of patients. The occurrence of liver tumors will aggravate malnutrition and may further weaken the antitumor and antitumor metastasis reaction [34, 35]. The total LY count can also reflect the patients' nutritional status and immune function. Malnutrition and low cellular immune function can decrease the total number of LYs [36]. The NLR is a systemic inflammation marker, and the early initiation of inflammation is a proinflammatory action mediated by macrophages, NEUs, and monocytes through releasing inflammatory factors [37]. In addition, a strong correlation between the PNI level and alcoholism history was identified, which was similar to the research of Chan et al. [38] who found that the preoperative PNI level was strongly correlated with the history of alcoholism in patients after LC surgery, while the NLR was significantly related to surgical treatment (with or without). As we all know, invasive surgery will affect the body's inflammatory state, resulting in postoperative complications, infections, etc. Zahorec et al. [39] showed that the gradual rise of LYs and the gradual decline of NEUs occurred simultaneously with the improvement of the clinical status of some major stress and systemic inflammatory responses. However, in our study, surgical treatment, with or without, was found to influence the NLR level.

The study also found a higher survival rate in the high-PNI group versus the low-PNI group and a lower survival rate in the high-NLR group versus the low-NLR group. Univariate and multivariate analyses identified the independence of the PNI and NLR as prognostic indicators for advanced HCC patients who received TT. The PNI and NLR have been indicated as independent prognostic indicators for HCC patients undergoing surgery [40, 41], as well as independent risk factors for early postoperative recurrence. Also, Gulmez A found that lower PNI (<38.25) values indicated lower PFS and rates in HCC [42]. The reason why the PNI can be used as a prognostic indicator for HCC patients may be because LYs are mainly involved in immune response, with the capacity of inhibiting tumor cell multiplication and metastasis [24]. The decrease in LY count, in contrast, weakens the systemic immune system, which allows the cancer cells to escape immunological surveillance easily, ultimately leading to an enhanced malignant phenotype of cancer cells. The serum ALB is the simplest and most efficient index that can reflect the body's nutritional condition and is the decisive factor in the immune response of cancer cells [43]. Hypoalbuminemia reduces the general immune system, resulting in tumour cell proliferation. Hence, the combination of LYs and serum ALB can better predict the outcomes of cancer patients. Furthermore, the NLR is one of the indicators reflecting immune status, which can be used to assess the body's antitumor inflammatory status. And, the NEUs can promote vascular endothelial growth factor secretion, inducing angiogenesis and tumour progression, thus predicting adverse clinical outcomes. Lymphocytosis is related to the immune escape of tumor cells, and the NEUs can inhibit the lethality of LYs [44]. Taken together, there are good reasons why the PNI and NLR can be used as independent predictors.

The present research has several limitations that need to be addressed. First, this is a retrospective, single-center study with limited cases included, requiring a large sample size to further validate the conclusions. Second, dividing the patients into two groups using data-derived cut-off values might lead to a decrease in statistical power and incomplete correction for confounding factors [45, 46]. Third, there are many factors that affect patient outcomes in addition to nutritional status and immune microenvironment, while many other biomarkers were not investigated because relevant data on the side effects of targeted therapies are lacking. Hence, a multicentric, large sample size, well-designed prospective study is needed to identify the efficacy and accuracy of the PNI and NLR for prognosis prediction of HCC patients receiving TT.

## 5. Conclusion

In conclusion, pretreatment PNI and NLR levels have great prognostic implications for advanced HCC patients undergoing TT. High NLR and Low PNI values suggest that this patient may have malnutrition and poor immune function, which is associated with an adverse prognosis. However, sorafenib monotherapy also shows great survival benefits in some patients. What matters is to identify biomarkers to predict which treatment a target patient will benefit most from. Using the markers in our study, it is possible to identify the patient population that is suitable for TT. We believe that such biomarkers can be used to identify suitable patients, especially in countries where not every patient has access to appropriate treatment for financial reasons. These tests have the advantage of being inexpensive, easy to calculate, and standardize.

## Figures and Tables

**Table 1:** Comparison of patients' clinicopathological features between the different PNI groups.

Indicators	Low-PNI group (*n* = 10)	High-PNI group (*n* = 73)	*χ* ^2^/*t*	*P*
Gender			2.2221	0.1362
Male	7 (70.0)	64 (87.7)		
Female	3 (30.0)	9 (12.3)		

Age			0.6349	0.4256
≤56	4 (40.0)	39 (53.4)		
>56	6 (60.0)	34(46.6)		

History of alcoholism			9.1451	0.0025
Yes	10 (100.0)	36 (49.3)		
No	0 (0)	37 (50.7)		

ECOG score			0.4017	0.5262
0-1	6 (60.0)	36 (49.3)		
2	4 (40.0)	37 (50.7)		

Child–Pugh classification			1.7111	0.1908
A	7 (70.0)	35 (47.9)		
B	3 (30.0)	38 (52.1)		

BCLC stage			0.2229	0.6368
B	4 (40.0)	35 (47.9)		
C	6 (60.0)	38 (52.1)		

Maximum tumor diameter			0.4017	0.5262
≤5 cm	4 (40.0)	37 (50.7)		
>5 cm	6 (60.0)	36 (49.3)		

Number of lesions			0.2625	0.6084
Single	3 (30.0)	28 (38.4)		
Multiple	7 (70.0)	45 (61.6)		

Surgery			0.6349	0.4256
Yes	6 (60.0)	34 (46.6)		
No	4 (40.0)	39 (53.4)		

*Note.* PNI, prognostic nutritional index; NLR, neutrophil/lymphocyte ratio; ECOG, Eastern Cooperative Oncology Group; BCLC, Barcelona clinic liver cancer. Bold text: statistically significant.

**Table 2:** Comparison of patients' clinicopathological features between the different NLR groups.

Indicators	Low-NLR group (*n* = 64)	High-NLR group (*n* = 19)	*χ* ^2^/*t*	*P*
Gender			0.0353	0.8509
Male	55 (85.9)	16 (84.2)		
Female	9 (14.1)	3 (15.8)		

Age			1.8671	0.1719
≤56	29 (45.3)	12 (63.2)		
>56	35 (54.7)	7 (36.8)		

History of alcoholism			0.5969	0.4398
Yes	34 (53.1)	12 (63.2)		
No	30 (46.9)	7 (36.8)		

ECOG score			0.1031	0.7481
0-1	33 (51.6)	9 (47.4)		
≥2	31 (48.4)	10 (52.6)		

Child–Pugh classification			0.1031	0.7481
A	33 (51.6)	9 (47.4)		
B	31 (48.4)	10 (52.6)		

BCLC stage			0.0014	0.9698
B	30 (46.9)	9 (47.4)		
C	34 (53.1)	10 (52.6)		

Maximum tumor diameter			0.9290	0.3351
≤5 cm	35 (54.7)	8 (42.1)		
>5 cm	29 (45.3)	11 (57.9)		

Number of lesions			0.2382	0.6255
Single	23 (35.9)	8 (42.1)		
Multiple	41 (64.1)	11 (57.9)		

Surgery			2.2101	0.1371
Yes	28 (43.8)	12 (63.2)		
No	36 (56.2)	7 (36.8)		

*Note.* PNI, prognostic nutritional index; NLR, neutrophil/lymphocyte ratio; ECOG, Eastern Cooperative Oncology Group; BCLC, Barcelona clinic liver cancer. Bold text: statistically significant.

**Table 3:** The short-term efficacy of patients in different PNI and NLR groups.

Groups	DCR			PD
	CR	PR	SD	
Low-PNI group (*n* = 10)	2 (20.0)	3 (30.0)	2 (20.0)	3 (30.0)
High-PNI group (*n* = 73)	22 (30.1)	24 (32.9)	7 (9.6)	20 (27.4)
*χ* ^2^/*t*	0.0122			
*P*	0.9121			
Low-NLR group (*n* = 64)	23 (35.9)	20 (31.3)	6 (9.4)	15 (23.4)
High-NLR group (*n* = 19)	1 (5.3)	7 (36.8)	3 (15.8)	8 (42.1)
*χ* ^2^	3.2131			
*P*	0.0731			

*Note.* PNI, prognostic nutritional index; NLR, neutrophil/lymphocyte ratio.

**Table 4:** Univariate regression analysis.

Prognostic factors	*β*	Se	Hazard ratio (95% CI)	*P*
Sex	0.234	0.356	1.263 (0.628–2.540)	0.512
Age	0.018	0.011	1.018 (0.996–1.040)	0.108
PNI	−1.565	0.308	0.209 (0.114–0.382)	<0.001
NLR	0.942	0.275	2.566 (1.498–4.397)	0.001
History of alcoholism	0.404	0.248	1.498 (0.921–2.437)	0.103
ECOG score	0.147	0.250	1.159 (0.710–1.890)	0.555
Child–Pugh classification	−0.142	0.247	0.867 (0.535–1.407)	0.564
BCLC stage	−0.226	0.250	0.798 (0.489–1.303)	0.367
Maximum tumor diameter	0.220	0.251	1.246 (0.762–2.037)	0.380
Number of lesions	0.263	0.259	1.301 (0.782–2.163)	0.311

Note. PNI, prognostic nutritional index; NLR, neutrophil/lymphocyte ratio; ECOG, Eastern Cooperative Oncology Group; BCLC, Barcelona clinic Liver Cancer. Bold text: statistically significant.

**Table 5:** Multivariate regression analysis.

Prognostic factors	*β*	SE	Hazard ratio (95% CI)	*P*
PNI	−1.415	0.306	0.243 (0.133–0.443)	<0.001
NLR	0.811	0.279	2.251 (1.304–3.885)	0.004

Note. PNI, Prognostic Nutritional Index; NLR, neutrophil/lymphocyte ratio. Bold text: statistically significant.

## Data Availability

The labeled dataset used to support the findings of this study are available from the corresponding author upon request.
